# Pediatrics

**Published:** 1900-04

**Authors:** Maud J. Frye

**Affiliations:** Buffalo, N. Y.


### Progress in Medical Science.

### PEDIATRICS.

Conducted by MAUD J. FRYE, M. D., Buffalo, N. Y.

### OTITIS MEDIA IN INFANCY.

POMEROY (*Boston Medical and Surgical Journal*, January 18, 1900,) emphasises the necessity for routine examination of the membrana tympani in pediatric practice. He quotes Ponfick, of Breslau, who in 100 consecutive autopsies on children under three years of age, dying from a variety of infections and noninfectious processes, found but nine children free from otitis media. He cites a series of interesting cases occurring in his own practice, illustrating the advisability of such examination. Regarding the technique of such examination the author says:

"The best artificial light, a head mirror and an assorted nest of specula are necessary. The external ear should be drawn downward and outward in the child, instead of upward and backward. If the external canal be obstructed by dirt or cerumen, it should be carefully washed. Nothing but long-continued practice will suffice to give one a definite idea of the appearance of the tympanic membrane, either in health or in disease, but with this practice any practitioner may become as expert as the most cultured aurist. In very many cases there is no abnormal appearance to the exterior of the tympanic membrane, and yet the middle ear may contain pus and be the source of toxemia, threatening life. An incision made carefully in the lower posterior quadrant of the tympanic membrane is wholly free from danger and will frequently reveal a case of grave disease, and at the same time be the means of almost instant relief. Thorough cleansing of the ear should be done before puncturing."

---

HYDROTHERAPHY IN PNEUMONIA.

BARUCH (*Pediatrics*, January 1, 1900,) says that in the pneumonic fevers of children I am in the habit of using the gradually reduced full bath, placing the child's entire trunk into water at 96°, using continuous friction over the body, while another person adds ice water, so as not to touch the body, until the bath water is reduced to 85 or 80°—duration five to eight minutes. Such a bath should be followed by drying and friction. I have found the chest compress at 60°, repeated every hour or two, an effective auxiliary. It deepens the inspirations; temporarily increases cough, and thus aids in the expulsion of stagnant secretions; arouses the heart, causing cyanosis to disappear for a time; and when the compress gets warm, we have the poultice effect of calming irritation, inducing sleep, and relieving pain, without the relaxation incident to the old-fashioned warm poultice. I usually begin the compress at 100°, then gradually reduce each time from one to five degrees, until sixty are reached.

In desperate conditions of broncho-pneumonia, whether idiopathic or complications of the exanthemata, when the skin is cyanotic, the respiration shallow, the pulse rapid and almost imperceptible, the extremities cold, although the internal temperature may be high, I am in the habit of ordering a stimulant and personally administering an affusion with a basinful of water at 60°, poured with some force over the shoulders of the child, while the latter is held semirecumbent in a tub of water at 105°, reaching to the navel. Followed by drying and friction, and repeated every hour or two, increasing the number of basins of water up to four, I have observed the overloaded bronchi relieved, the inspiration deepened and calmed, expectoration increased, cyanosis removed, and the pulse greatly improved.

### THE IMPORTANCE OF REST AFTER ACUTE CARDIAC INFLAMMATIONS IN CHILDREN.

HOLT (*Archives of Pediatrics*, December, 1899,) emphasises the importance of prolonged rest in bed after acute cardiac inflammations in children. He says: There are three reasons why cardiac inflammations are likely to be especially serious in young children. (1) The frequency with which both the endo- and pericardium are involved; (2) the great tendency to acute dilatation; (3) the liability of these attacks to be complicated by pneumonia. The cardiac muscle, like the voluntary muscles, in young children, has by no means the resistance which it attains in later life; as a consequence of this, it happens that under the strain of an acute inflammation, dilatation comes on very readily in the adult. The danger of dilatation is much increased in cases complicated by pericarditis. It is largely owing to rapid dilatation, that we see so many cases succumb during the acute attacks, and it is usually in consequence of progressive dilatation that cardiac failure with dropsy and all its attendant features so often follows within a few months of the primary attack.

This condition of the heart walls must be the chief consideration in the treatment of acute attacks, not only during the period of active inflammations, but for a considerable time afterward. If we would minimise the injurious effects of acute endo-pericarditis, we must secure to these patients as nearly absolute rest as is possible, for a period of two or three months after acute symptoms have subsided. In few things are physicians more blameworthy than in allowing children to get up as soon as acute symptoms have disappeared, after they have suffered either from acute cardiac inflammations or from general infectious diseases, particularly diphtheria, scarlet fever or typhoid. Two cases illustrating the possibilities of cure when prolonged rest is carried out, and one the disastrous results following failure to adopt the rest treatment, are reported in detail.

---

### THE PREVENTION OF FEEBLE-MINDEDNESS.

AT LEAST as much importance (*Brit. Med. Jour.*, 1899, No. 2026, 1209,) attaches to the means of preventing mental feebleness as to the treatment of defective children. When myopia and adenoids—which not uncommonly occur together—are neglected, children are apt to be mentally backward in their school life; neglect of treatment leads to imperfect appreciation through the senses, resulting in deficient mental training. Crippled children may have good brains, though their physical defects prevent the ordinary means of education, which need to be adopted under the circumstances. Choreic children who have had several such illnesses are often delicate, but are not usually exceptionally dull if the necessary advantages are afforded them. In times past it was often thought necessary to leave the epileptic children without education; modern experience has shown that full, healthy occupation, which may be made educative, increases brain stability and tends to save mental power; while, when the fits begin to pass off, systematic education under due supervision may prevent that degree of mental inaptitude and irritability which so often follows epilepsy. Even the imbecile infant, if well trained from the earliest days, may become well behaved, of good habits, and social appearance; whereas, when neglected, he is apt to grow up unsocial and a greater burden to others than needs be. In all cases of congenital defect early diagnosis and good management are necessary.—*Pediatrics.*

---

THE BATH IN TYPHOID FEVER.

A. Jacobi (*Pediatrics*, December 15, 1899,) has the following to say concerning bathing, in an article on Typhoid Fever in the young:

"Cold water and warm water are our most reliable and at the same time the safest antipyretics. Stress should be laid on the latter title, because many of the very apostles of hydrotherapy, perhaps influenced by shaky phagocytosis and toxin theories, belittle it in comparison with the nerve stimulating powers of water. Now, cold bathing is frequently contraindicated; it is not borne when the heart is feeble from whatsoever cause; for instance, long duration of the disease, complications with pneumonia, peritonitis, or hemorrhages, previous bad health, or, in the adult, excesses. No stimulant given before or during the procedure is certain to counteract the paralyzing effect on the peripheral circulation. When after a cold bath the feet remain cold and the pulse small, the bath was contraindicated, and did harm. The patients in public hospitals are quite often of a low vitality, and feel the cold bath as a shock; at all events, most of those who arrive in the hospital after a week or two have passed the time when the cold bath might have done good. Of this nature is the latest experience of I. Rudisch in the Mount Sinai Hospital (Mt. S. Rep., Vol., 199). He says: "The Brand treatment reduced the mortality a little over 2 per cent. This reduction occurred in the cases which had been sick two weeks or longer, outside the hospital. Since the introduction of the Brand treatment there has been an increase in the number of cases of pneumonia and phlebitis, and a decrease of those of furunculosis and nephritis. Relapses have increased 2.5 per cent. The death-rate in the relapse cases before and since the introduction of the Brand treatment is practically the same. It has not reduced the number of complicated cases as a whole, but has decreased the number of deaths from toxemia in the causation of the mortality of typhoid fever."

The dangers of cold bathing are not encountered in warm bathing. This is not the place to prove for the thousandth time that a bath of 95 or 90° F. when of sufficient, even when of short duration, will reduce a temperature of 104 or 106°. It is simply a fact. Such a bath may easily be given every three or five hours; even apparently mild cases should have two or three daily, from the beginning to the end of the illness. They reduce the temperature, the accompanying frictions stimulate the cutaneous and general circulation, the general condition is improved, the so-called typhoid state relieved, and the relapses become less frequent. Warm bathing should be the principal treatment of all typhoid fevers, not to the exclusion, however, of occasional medication calculated to have similar effects. The combination of frequent and protracted bathing with proper medication will always remain appropriate, though our resources should, as we expect, be increased by sero-therapy.

